# Positive Feedback Regulation between Transglutaminase 2 and Toll-Like Receptor 4 Signaling in Hepatic Stellate Cells Correlates with Liver Fibrosis Post *Schistosoma japonicum* Infection

**DOI:** 10.3389/fimmu.2017.01808

**Published:** 2017-12-13

**Authors:** Zhencheng Wen, Xiaofang Ji, Juanjuan Tang, Guiying Lin, Linzhuo Xiao, Cuiying Liang, Manni Wang, Fang Su, Dominique Ferrandon, Zi Li

**Affiliations:** ^1^Sino-French Hoffmann Institute, Guangzhou Medical University, Guangzhou, China; ^2^Université de Strasbourg, RIDI UPR9022 du CNRS, Strasbourg, France

**Keywords:** toll-like receptor 4 signaling, transglutaminase 2, liver fibrosis, hepatic stellate cells, *Schistosoma japonicum*

## Abstract

Liver fibrosis induced by *Schistosoma japonicum (Sj)* infection is characterized by the accumulation of extracellular matrix (ECM). The activated and differentiated hepatic stellate cells (HSCs) are the predominant ECM-producing cell type in the liver. Toll-like receptor (TLR) 4 pathway activation plays a key role in mice liver fibrosis models induced by alcohol, biliary ligation, and carbon tetrachloride 4. In this work, we found that TLR4 pathway activation correlated with the severity of liver fibrosis post *Sj* infection. The TLR4 receptor inhibitor TAK242 reduced the extent of liver fibrosis. The increased expression of TLR4, α-smooth muscle actin (α-SMA), and cytoglobin was observed in the HSCs of mouse liver after *Sj* infection. In response to stimulation with either lipopolysaccharide or *Sj’s* soluble egg antigen (SEA), high levels of TLR4 and α-SMA were induced in HSCs and were inhibited by TAK242 treatment. In previous work, we had reported that a high level of transglutaminase 2 (TGM2) is crucial for liver fibrosis post *Sj* infection. Herein, we found that TLR4 signaling also controlled Tgm2 expression. Inhibition of TGM2 activity by cystamine (CTM) in *Sj*-infected mice or in HSCs induced with all-trans-retinoic acid (ATRA) stimulation led to a lowered activation of TLR4 signaling and a reduced α-SMA expression. These results were confirmed by downregulating the *Tgm2* gene by specific siRNA. These observations implied the presence of a positive feedback regulation between TGM2 and TLR4 signaling in HSCs that correlated with liver fibrosis post *Sj* infection. This novel connection between TGM2 and TLR4 pathway activation in liver fibrosis induced by *Sj* infection enhances our understanding of liver diseases.

## Introduction

*Schistosoma japonicum (Sj)* infection has been reported in 12 provinces in China to date (1). *Sj*-infected hosts display serious pathological changes in the liver. Hepatic fibrosis develops as a response to chronic liver injury or infection and occurs almost exclusively in a pro-inflammatory environment. However, the role of inflammatory mediators in the fibrogenic responses of the liver remains poorly understood. The activation and proliferation of hepatic stellate cells (HSCs), which are characterized by the morphological transition to myofibroblast-like cells marked by a high level of α-SMA expression and extracellular matrix (ECM) deposition [e.g., collagen type I (COL I) and III (COL III), encoded, respectively, by the col1a1 and col3a1 genes] have been well established as central events in the pathogenesis of hepatic fibrosis (2, 3). HSCs are the predominant ECM-producing cell type in the liver (4). Previous studies suggest that inhibiting the activation, proliferation, and migration of HSCs may be an attractive option for anti-fibrotic therapy (4). The inflammatory mechanisms that underlie the activation of HSCs and the liver fibrosis induced by *Sj* infection both warrant further study.

The toll-like receptors (TLRs) in liver tissues are expressed on Kupffer cells, endothelial cells, dendritic cells (DCs), biliary epithelial cells, HSCs, and hepatocytes. TLRs, especially TLR4 signal pathway, play critical roles in liver fibrosis (5–7). A deficiency in TLR4 expression attenuates alcoholic or non-alcoholic steatohepatitis in mice (8–10), which is similar to liver fibrosis induced in mice by biliary ligation and exposure to carbon tetrachloride 4 (11). HSCs express all TLRs in the liver, including TLR4 (12, 13). TLR4 signaling can be activated in the HSCs of mice with liver fibrosis induced by biliary ligation (11). Lipopolysaccharide (LPS) is the major ligand of TLR4. In response to LPS stimulation, some inflammatory cytokines and chemokines are upregulated in HSCs, including intercellular cell adhesion molecule-1 (ICAM-1), vascular cell adhesion molecule 1 (VCAM-1), chemokine (C–C motif) ligand 5 (CCL5), and chemokine (C–X–C motif) ligand 10 (CXCL10), most of which can directly activate HSCs (11, 14, 15). The glycolipids in *Schistosoma mansoni (Sm)* adult worm, the C-type lectin DC-specific intercellular adhesion molecule-3-grabbing non-integrin (DC-SIGN), and TLR4 on DCs have crucial roles in DC activation, which can skew the T cell response toward a Th1 profile during the early stage of a *Sm* infection (16). However, no study has examined the relationship between the TLR4 signal pathway and *Schisotosoma*-induced fibrosis.

Transglutaminase 2 (TG2 or TGM2), also known as tissue transglutaminase, is a multifunctional enzyme involved in several important biological processes, including cell death, signaling, cytoskeleton rearrangements, ECM stabilization, and fibrosis (17, 18). We have provided evidence that TGM2 regulates the TGF-β1 of parasite origin and IL-13 of the host as well as documented the important role of TGM2 in liver fibrosis during *Sj* infection (19, 20). Tgm2 expression has been reported to be induced by LPS in macrophages, microglial cells, and astrocytes (21–23). The genetic knockout of Tgm2 confers resistance to LPS-induced septic shock in mice and has been associated with the capacity to restore the initial equilibrium of circulating cytokines and pro-inflammatory mediators (24). On the other hand, a marked downregulation of membrane TLR4 in DCs was observed in Tgm2-knockout mice (25). However, the relationship among TLR4 signaling, TGM2, and liver fibrosis in HSCs has never been reported in the literatures.

Based on above findings, the objectives of this study were to gain further insights into the mechanisms that lead to the dysregulation of HSCs activation in liver fibrosis post *Sj* infection. We specifically focused on the possible role of TLR4 signaling, TGM2, and their feedback regulation.

## Materials and Methods

### Reagents

Sirius red staining kit was purchased from Abcam (USA). The antibodies used were as follows: anti-α-SMA (BS70000, Bioworld Technology, China), anti-TLR4 (Ab47093, Abcam, USA), anti-COL I (14695-I-AP, Abcam, USA) and anti-COL III (Ab7778, Abcam, USA), anti-TMG2 (3557, Santa Cruz Biotechnology, USA), anti-cytoglobin (CYGB) (Ab52662, Abcam, USA), anti-desmin (DM0095, Boster, China), anti-cleaved-Caspase3 (9664, CST, USA), anti-p-IRAK1 (bs-3194R, Bioss, China), anti-β-Tubulin (DKM9003, Sanjian Biotechnology, China), and HRP-conjugated secondary antibodies of mice or rabbit IgG (SA00001, Proteintech, Wuhan, China), Alexa Fluor-488- and Alexa Fluor-594-conjugated anti-mouse and anti-rabbit secondary antibodies (35552 and 35510, Invitrogen, USA). Lipopolysaccharide, a ligand of TLR4, was purchased from Sigma-Aldrich (USA). TAK242, a small-molecule inhibitor of the TLR4 receptor that blocks binding to downstream adaptors in the signal pathway, was purchased from Shanghai Haoyuan Chemexpress. Cystamine (CTM), the inhibitor of TGM2 enzyme activity, was obtained from Sigma-Aldrich (USA). Bicinchoninic acid protein assay kit was purchased from the Beyotime Institute of Biotechnology (Jiangsu, China). 3,3′-diaminobenzidine substrate kit was purchased from Gene Tech Company Limited (Shanghai, China). Hoechst33342 was purchased from Sigma-Aldrich (USA), while the powder of the soluble egg antigen (SEA) of *Sj* was obtained from the National Institute of Parasitic Diseases in Chinese Center for Disease Control and Prevention. PrimeScript^®^ RT Reagent Kit with gDNA Eraser (Perfect Real Time) and SYBR^®^ Premix Ex Taq™ II (Tli RNase H Plus) were purchased from TaKaRa Biotechnology Co., Ltd. (Dalian, China). Trizol was obtained from Life Technologies (USA), and all-trans-retinoic acid (ATRA) was obtained from Sigma-Aldrich (USA). Recombinant human TGM2 protein [recombinant TGM2 (rTGM2), endotoxin level <1.0 EU per 1 µg] was purchased from R&D systems (USA). Lipofectamin RNAiMAX RNAi transfection reagent was purchased from Invitrogen (USA). The Tgm2 siRNAs targeting the 3-UTR of the Tgm2 mRNA were synthesized by Robibio Biotechnology Limited. The target sequences are as follows: Tgm2 siRNA-1, 5′-CCTTCTCATCGAGTACTTC-3′; Tgm2 siRNA-2, 5′-TCAATGCCGACGTGGTAGA-3′; Tgm2 siRNA-3, 5′-GCCTGATCCTTCTAGATGT-3′.

### Mice, Ethics Statement, Infection of Parasites, and Treatments

Six- to eight-week-old female BABL/c mice (Experimental Animal Center of Sun Yat-Sen University, Guangzhou, China) were maintained according to the guidelines and were approved to be appropriate and humane by Institutional Animal Care and Use Committee of Guangzhou Medical University. These mice were fed with commercially available diet and housed in a controlled environment at 23 ± 2°C. They were infected percutaneously through the abdomen with 20 ± 3 *Sj* cercariae of Chinese mainland strain. The mice developed liver granuloma and then acute, advanced, and chronic fibrosis at weeks 5, 6, 8, and 12 after they were infected (*n* = 8–10), respectively. The uninfected mice were used as normal controls (*n* = 7). Three days post-infection, the mice group with TGM2 inhibitor–CTM treatment (*n* = 8–10) were administered with 100 µl CTM (10^−2^ M) in PBS by intraperitoneal injection once a day for 7 days (26), while the infection control group (*n* = 7) only received PBS. Two non-infection control mice groups were treated with CTM (*n* = 7) and PBS (*n* = 7), respectively. Four weeks post-infection, the mice group with TLR4 pathway inhibitor–TAK242 treatment (*n* = 8–10) were administered with 100 µl TAK242 (0.3 mg/kg) in PBS by intraperitoneal injection twice a week for 4 weeks, while the infection control group (*n* = 7) only received PBS. Two non-infection control mice groups were treated with TAK242 (*n* = 7) and PBS (*n* = 7), respectively. All mice were sacrificed at week 8 and their liver tissues and sera were collected for further detection.

### Measurement of the Severity of Liver Fibrosis in Mice *via* Sirius Red Staining

Fresh liver tissues were fixed in 4% paraformaldehyde for 24 h and routinely embedded with paraffin. Liver sections of 5 µm were then prepared for Sirius red staining to evaluate the degree of liver fibrosis. The area of morphometric collagen as shown in red was analyzed using the Image J software. Each stained sample was evaluated in a double-blind fashion by two independent researchers.

### RNA Isolation and Quantitative PCR

The total RNA from fresh liver tissues and HSCs was extracted with a Trizol reagent, and cDNA was synthesized using PrimeScript^®^ RT Master Mix according to the instructions of the manufacturer. cDNA was amplified by quantitative real-time PCR(qPCR) using the SYBR Green I PCR master kit and the CFX96™ real-time system (BIO-RAD, USA). Table 1 lists the primer sequences. The mRNA level of individual genes was normalized to GAPDH and calculated using the 2^−ΔΔCt^ data analysis method.

**Table 1:** Primer sequences used in qPCR.

Proteins	Genes	Forward primer sequence	Reverse primer sequence
**Mice**			
GAPDH	Gapdh	TGTGTCCGTCGTGGATCTGA	TTGCTGTTGAAGTCGCAGGAG
α-SMA	Acta2 (actin, alpha 2, smooth muscle, aorta)	TTCGTGTGGCCCCTGAAGAGCAT	CCAGTTGTACGTCCAGAGGCA
Col I	Col1a1	CGTAAGCACTGGTGGACAGA	TCTGAGGAATGCCAGCTGCA
Col III	Col3a1	AGCACGTCTGGTTTGGAGAG	GACATTAGGCGCAGGAAGGT
TLR4	Tlr4	ACCTGGCTGGTTTACACGTC	CTGCCATATACATTGCAGAA
CD14	Cd14	ATTTGCATCCTCCTGGTTTC	AAATCAGGGGTCAAGTTTGC
Pro-IL-1β	Il1b	TTGACGGACCCCAAAAGATG	TGGACAGCCCAGGTCAAAG
TNF-α	Tnf	CAGGAGGGAGAACAGAAACTCCA	CCTGGTTGGCTGCTTGCTT
CXCL10	Cxcl10	CTGCCGTCATTTTCTGCCTC	TTCAAGCTTCCCTATGGCCC
CCL5	Ccl5	ATATGGCTCGGACACCACTC	TCTTCTCTGGGTTGGCACACA
IFN-α	Ifna1	TTTCCCCTGACCCAGGAAGATG	CTCTCAGTCTTCCCAGCACATT
Transglutaminase 2 (TGM2)	Tgm2	CTAGAGGCTTCTACTGGCTACC	GCGTAAGGACATATTCCCGTC
Fibronectin 1 (FN1)	Fn1	CTACGGAGAGACAGGAGGAAATAGC	AGTGACAGCATACAGGGTGATGG

**Humans**			
GAPDH	GAPDH	GAACGGGAAGCTCACTGG	GCCTGCTTCACCACCTTCT
Pro-IL-1β	IL1B	ATGATGGCTTATTACAGTGGCAA	GTCGGAGATTCGTAGCTGGA
CXCL10	Cxcl10	GTGGCATTCAAGGAGTACCTC	TGATGGCCTTCGATTCTGGATT
IL-6	IL6	TCCACAAGCGCCTTCGGTCCAGTTG	AGAGGTGAGTGGCTGTCTGTGTGGG

### Semi-Quantitative Immunohistochemical (IHC) Assay

After sacrificing the mice, their livers were quickly fixed in 4% paraformaldehyde overnight before they were dehydrated with alcohol and embedded with paraffin. Liver sections of 5 µm were prepared. Endogenous peroxidase was blocked with 3% hydrogen peroxide (H_2_O_2_) and nonspecific background was eliminated through a 10% bovine serum albumin treatment. The tissue sections were then incubated with specific primary antibodies, including anti-: α-SMA (1:400), COL I (1:400), COL III (1:400), p-IRAK1(1:400), or desmin (1:400), followed by HRP-conjugated secondary anti-rabbit or anti-mouse antibodies. The images were observed and captured with an optical microscope (Olympus, Japan).

Five fields were randomly selected from each section of one sample at 400× magnification, and the semi-quantitative IHC assay was determined based on the modified H-score (27). The results of the IHC assay were scored in a semi-quantitative fashion by incorporating both the staining intensity level and the specific positive staining area (400×). Three staining intensity levels and five categories for the percentage of positive staining areas were scored as shown in Table 2. The score of each field from the same section was computed by multiplying a weighted staining intensity level by the percentage of positive staining area. The final score (H-score) of each tissue section was equal to the average score of five fields ranging from 0 to 12.

**Table 2:** Scoring parameters of semi-quantitative Immunohistochemical.

Staining intensity level	Score	Percentage of positive staining area (%)
–	0	0–5
Canary yellow	1	6–25
Pale brown	2	26–50
Tan	3	51–75
–	4	76–100

The protein expression level in IHC was standardized based on H-score as follows: 0: negative expression or (+); 1–4: weak expression or (++); 5–8: moderate expression or (+++); and 9–12: strong expression or (++++). The semi-quantitative level of target protein expression in each group was determined based on the average H-score among the sections of different samples.

### Semi-Quantification of the Single or Double-Positive Cells for α-SMA and TLR4 Immunofluorescence (IF)

The liver sections of *Sj*-infected mice with or without TAK242 or CTM treatment were incubated with primary antibodies against α-SMA or CYGB and TLR4 at 4°C overnight. Afterward, these sections were incubated with Alexa Fluor-488- and Alexa Fluor-594-conjugated anti-mouse and anti-rabbit antibodies (1:4,000) for 1 h at room temperature in the dark. Cell nuclei were stained with Hoechst 33343 (1:4,000) for 30 min. The slides were then mounted with an antifading medium. Immunofluorescent images were observed and captured using a Zeiss LSM Pascal Axiovert confocal microscope (Carl Zeiss). The number of single TLR4 or α-SMA or CYGB positive cells as well as α-SMA or CYGB and TLR4 double-positive cells in each field at 400× magnification was calculated using Image J. Five images were randomly selected from each sample, and the staining intensity of the selected images was quantified with a preselected threshold using Image J.

### *In Vitro* Cultured Human HSCs Line

A human HSCs line (LX-2) was obtained from the Shanghai Cell Institute (Shanghai, China) and was maintained in a DMEM culture medium with 10% fetal bovine serum, 1 mmol/L glutamate, 100 U/mL penicillin, and 100 µg/mL streptomycin.

### Western Blotting

The proteins from mouse livers and the cultured HSCs line were extracted in radio-immunoprecipitation assay buffer. Equal amounts of cell proteins were resolved in 10% SDS-polyacrylamide gels and then transferred to PVDF membranes. After incubating with the primary and secondary antibodies, the target proteins were visualized using an enhanced ECL reagent according to the instructions of the supplier.

### RNA Interference

Hepatic stellate cells were seeded in a six-well plate the day before transfection at 30–50% confluency. Tgm2 siRNA was transfected with Lipofectamin RNAiMAX RNAi transfection reagent. 48 h after transfection, TGM2 protein levels were measured by Western blotting.

### Statistical Analysis

The results are presented as the mean or SEM (±SD) of the indicated number of replicates/experiments. We performed one-way analysis of variance to calculate the statistical differences among multiple groups, and then performed paired comparisons through *t*-test. An adjusted *P*-value of ≤0.05 was considered statistically significant.

## Results

### Activation of the TLR4 Signal Pathway Correlates with the Extent of Liver Fibrosis Post *Sj* Infection

**Figure 1:**
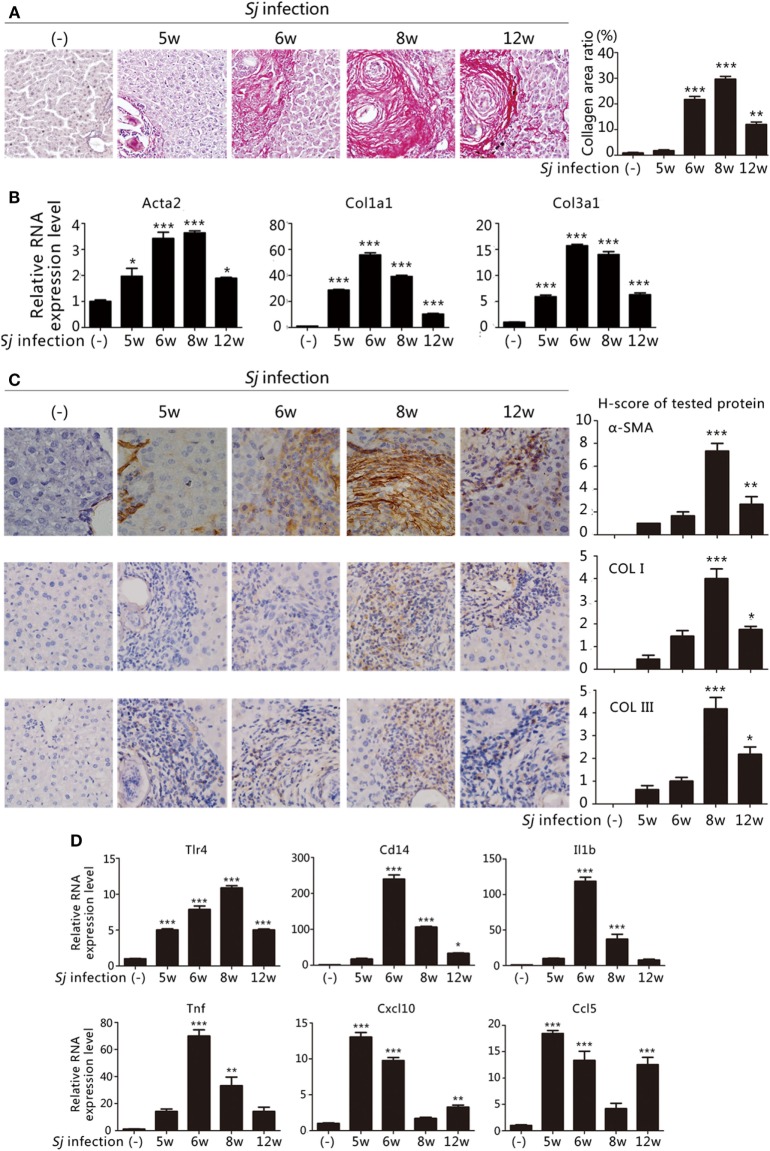
Toll-like receptor (TLR) 4 pathway activation correlates with the extent of liver fibrosis post *Schistosoma japonicum* (*Sj)* infection. BALB/c mice were infected with 20 ± 3 infective cercariae of *Sj* for 5, 6, 8, and 12 weeks, and non-infected mice served as negative control. Liver tissues were fixed and stained with sirius red. A typical staining (200×) is shown in [**(A)** *left*]. Percentage of the total areas of morphometric collagen displayed with red color was calculated and shown in [**(A)** *right*]. **(B)** The steady-state mRNA expression levels of Acta2, Col1a1, and Col3a1 in the liver tissue of BALB/c mice were measured by quantitative-RT-PCR (qPCR). Gapdh was used as an internal reference; **(C)** The protein expression levels of α-SMA, COL I, and COL III in the mouse liver tissue were determined by immunohistochemical (IHC) assay (400×, *left*), and the semi-quantitative level of these proteins was analyzed using a modified H-score procedure (*right*); **(D)** The steady-state mRNA expression levels of TLR4 signal pathway-related molecules (Tlr4, Cd14, Il1b, Tnf, Cxcl10, and Ccl5) in the mouse liver tissue were measured by qPCR. Data are presented as mean ± SD from 7–10 mice per group. All experiments were performed twice. *P ≤ 0.05; **P < 0.01; ***P < 0.001.

The extent of liver granuloma and fibrosis was evaluated by monitoring the liver aspect, collagen deposition (Sirius red staining) (Figure 1A), and HSCs activation [high RNA and protein expression levels of Acta2 (which encodes α-SMA), Col1a1, and Col3a1] (Figures 1B,C). The week-8 *Sj* infection group displayed the largest collagen deposition area (29.66 ± 1.07%), followed by the week 6 (21.69 ± 1.24%), week 12 (11.98 ± 0.95%), and week 5 (1.76 ± 0.34%) groups (Figure 1A, *right*). The levels of Acta2, Col1a1, and Col3a1 transcription and translation in the liver increased after *Sj* infection at weeks 5, 6, 8, and 12, and peaked at week 8 (Figure 1B). The expression of α-SMA, COL I, and COL III was moderate at week 8 after *Sj* infection (Figure 1C, *right*). Consistent with the results of our previous study (19, 20), liver granuloma induced by *Sj* infection began at week 5, acute fibrosis began at week 6, and advanced liver fibrosis began at week 8. The areas of collagen deposition and the expressions of α-SMA, COL I, and COL III all decreased at week 12 (chronic liver fibrosis).

To examine the correlation between TLR4 signaling and liver fibrogenesis, qPCR was used to screen the transcriptional profiles of hepatic Tlr4 and Cd14, which encode, respectively, transmembrane and membrane associated receptors, Tnf and Il1b that code for effector cytokines of MyD88-dependent TLR4 pathway activation, and Cxcl10 and Ccl5 chemokine genes, which are used as readouts of MyD88-independent TLR4 pathway activation. The mRNA levels of Tnf and Il1b significantly increased after *Sj* infection at week 6 during acute liver fibrosis and subsequently remained high at the advanced liver fibrosis stage (week 8). The level of Cxcl10 and Ccl5 strongly increased at weeks 5 and 6 when the *Sj* eggs began to induce granuloma and acute liver damage, and no differences were observed between week 8 and the non-infected control (Figure 1D). These data are compatible with the hypothesis that TLR4 pathway activation triggers the host inflammatory response during *Sj* infection.

### Inhibition of TLR4 Signaling Alleviates Hepatic Fibrosis Induced by *Sj* Infection

**Figure 2:**
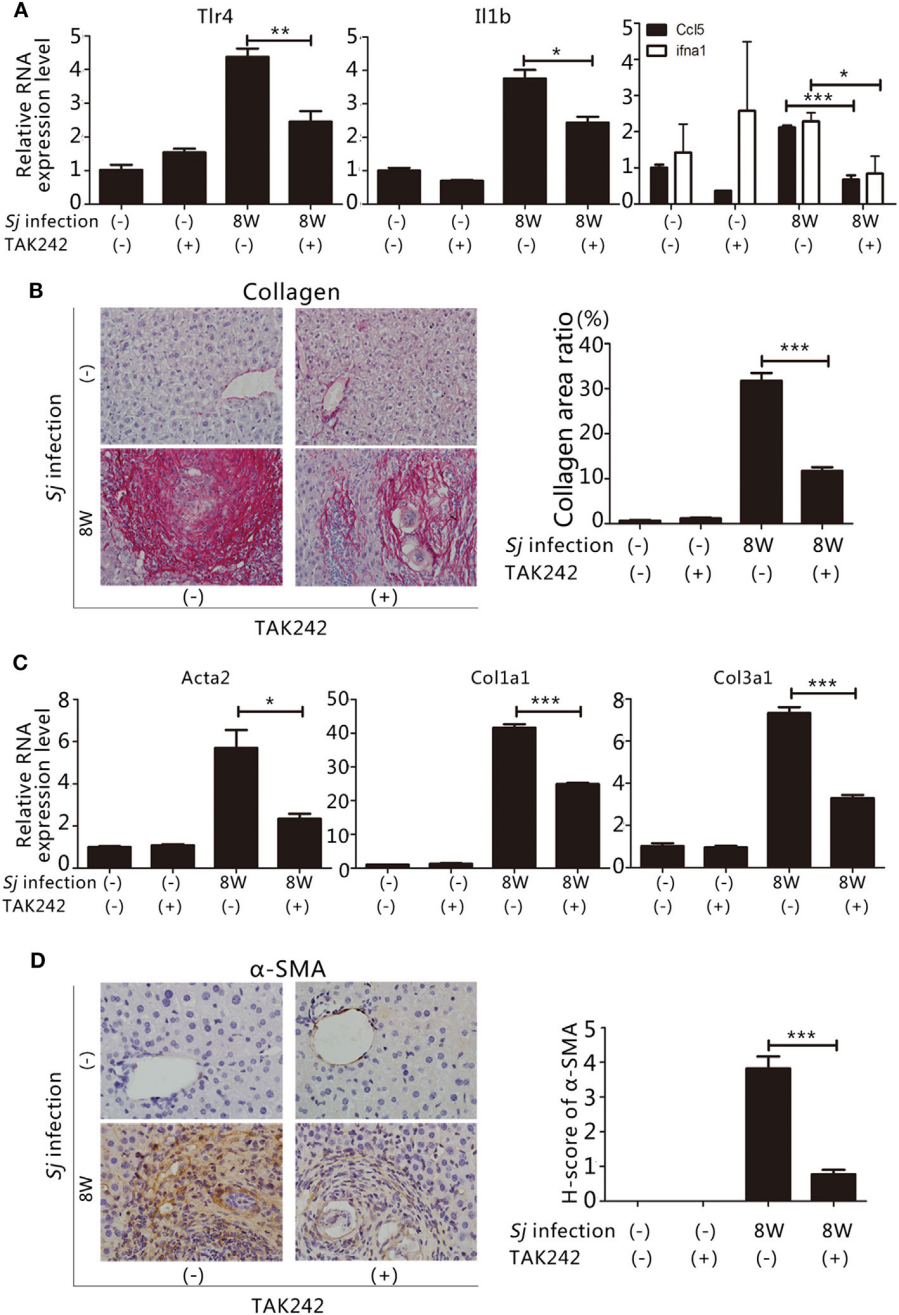
Suppression of toll-like receptor (TLR) 4 signaling by TAK242 treatment decreases the severity of hepatic fibrosis post *Schistosoma japonicum* (*Sj*) infection. TLR4 signal pathway in BALB/c mice was blocked by intraperitoneal injection of TAK242 from week 4 to 8 post *Sj* infection. Non-infected mice with or without TAK242 treatment were used as controls. **(A)** The steady-state mRNA expression levels of Tlr4, Il1b, and ifna1, Ccl5 in the mouse liver were evaluated by qPCR to assess the inhibitory effect of TAK242 on TLR4 signaling. Gapdh was used as an internal control. Data are presented as mean ± SD from 7 to 10 mice per group; **(B)** A typical sirius red staining section (200×) of a mouse liver section is shown on the left panel. The semi-quantitative level of collagen deposition is shown on the right panel; **(C)** The steady-state mRNA expression levels of Acta2, Col1a1, and Col3a1 in the liver tissue were determined by qPCR; **(D)** A typical immunohistochemical (IHC) staining section (400×) for α-SMA, COL I, and COL III in the liver of BALB/c mice is shown on the left panel, and the modified H-score results are shown on the right panel. Data are presented as mean ± SD. All experiments were performed twice. *P ≤ 0.05; **P < 0.01; ***P < 0.001.

To assess whether the TLR4 signal pathway is involved in the formation of liver fibrosis induced by *Sj* infection, we started to inject the TLR4 signal pathway inhibitor TAK242 (28) to *Sj-*infected mice at week 4 of infection. The whole treatment lasted for 4 weeks. TAK242 treatment lowered the mRNA levels of Tlr4, Il1b, and ifna1, Ccl5 (Figure 2A). In addition, the TAK242-treated mice exhibited a significant reduction in their collagen deposition area percentage after *Sj* infection (Figure 2B). The mRNA and protein expression levels of Acta2, Col1a1, and Col3a1 (Figures 2C,D) of these mice were lower than those of TAK242-untreated mice. The expression of α-SMA changed from moderate to low level according to H-score evaluation in the IHC assay (Figure 2D). TAK242-treated mice showed a significant decrease in parasite egg number after *Sj* infection compared to the non-treated mice (*P* ≤ 0.05, Figure S1 in Supplementary Material). These findings suggest that inhibiting TLR4 signaling alleviated the egg load and extent of liver fibrosis, thereby highlighting TLR4 pathway activation as an important pathogenic mechanism that induces liver fibrogenesis post *Sj* infection.

### TLR4 and α-SMA Expression Is Induced in *Sj*-Infected Liver HSCs, and LPS, and *Sj* SEA Triggered the Activation of *In Vitro* Cultured HSCs

**Figure 3:**
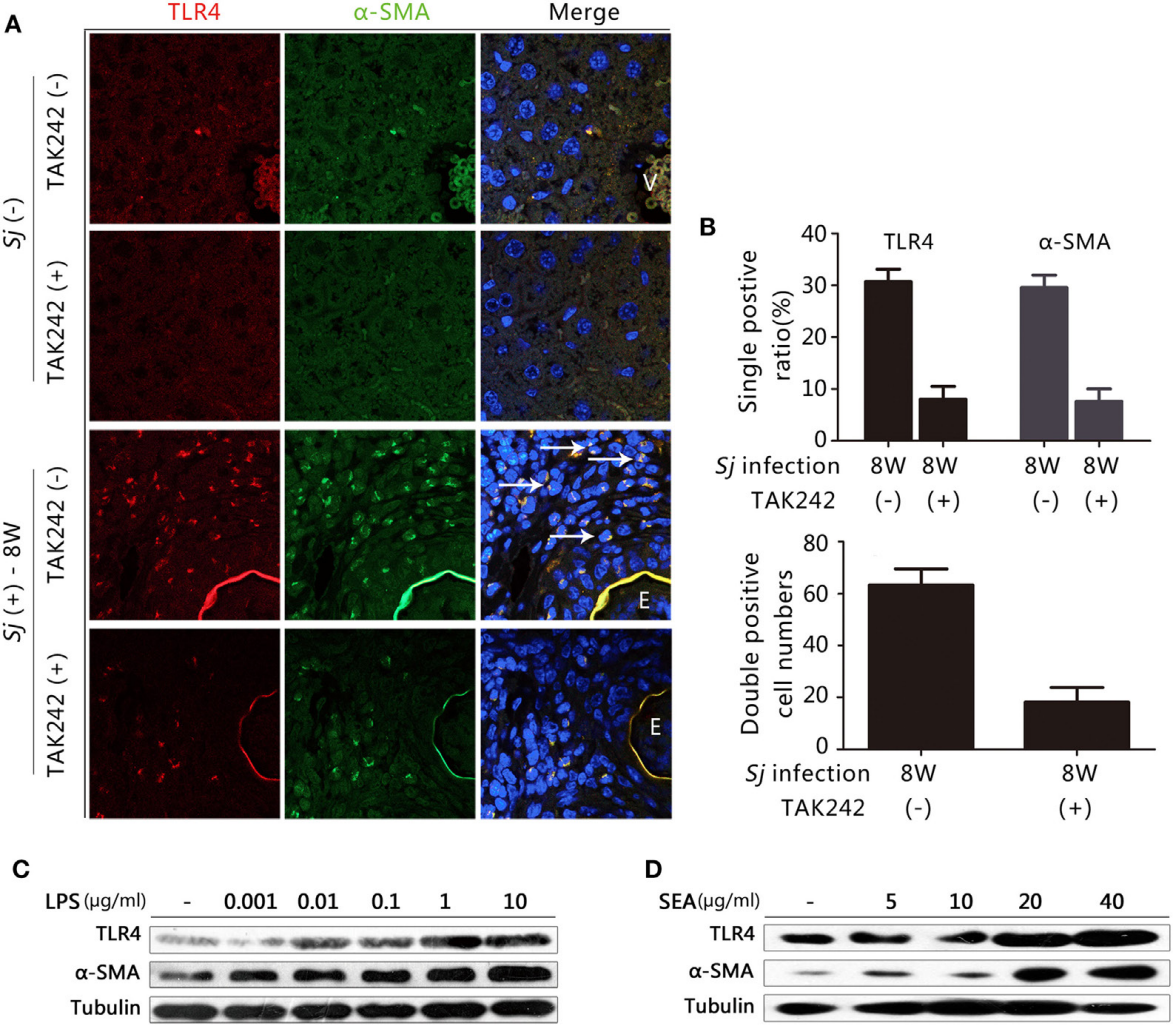
Correlation between toll-like receptor (TLR) 4 and α-SMA expression in *Schistosoma japonicum* (*Sj*)-infected livers, and after exposure of cultured hepatic stellate cells (HSCs) to lipopolysaccharide (LPS) or *Sj* soluble egg antigen (SEA). **(A)** The expression of TLR4 and α-SMA was revealed by immunofluorescence (IF) staining (400×) in a mouse liver section of BALB/c mice post *Sj* infection with or without TAK242 treatment. A typical IF result is shown. **(B)** The positive expression ratio of TLR4 or α-SMA single positive cells in the total cells of mouse liver sections is shown at 200× magnification (top), and the total numbers of cells with TLR4 and α-SMA doubly positive were calculated and shown (bottom). **(C,D)** TLR4 and α-SMA protein levels of cultured HSC [with or without LPS **(C)** or *Sj* SEA **(D)** stimulation in different doses] were measured by western blotting. β-Tubulin was used as a loading control. All experiments were performed at least twice. The arrows pointed to TLR4 and α-SMA double-positive cells. E, Eggs of *Sj*; V, vein in the mice liver.

Hepatic stellate cells activation plays a crucial role in the progression of hepatic fibrosis (2, 3). TLR4 signaling in HSCs, but not in Kupffer cells, is crucial for the development of liver fibrosis induced by alcohol, CCL4, and viral infection (11, 13). In humans, CYGB has been reported to be a relevant marker for distinguishing stellate cells from portal myofibroblasts in the damaged liver, while α-SMA marks activated HSCs and myofibroblasts (29). However, in mice, α-SMA and desmin are used as markers for activated and total HSCs, respectively (30). We explored whether the TLR4 signal pathway is activated in HSCs during *Sj* infection. In the week 8 *Sj-*infected liver but not in the non-infected control, TLR4 (red fluorescence) was highly expressed in the cells surrounding the vessels where *Sj* adult worms located. TLR4 was especially expressed in the granuloma where *Sj* eggs are deposited, while α-SMA (green) (Figure 3A) or CYGB (green) (Figure S2 in Supplementary Material, *left*) was highly expressed in HSCs or appeared as fiber-like materials surrounding the eggs. Consistent with the results of qPCR and IHC (Figure 2), TAK242 decreased the levels of TLR4, α-SMA (Figure 3B, *top*), and CYGB (Figure S2 in Supplementary Material, *right top*) in the liver 8 weeks after *Sj* infection. As expected, TLR4 and α-SMA or TLR4 and CYGB doubly positive cells were found, and TAK242 treatment did decrease the number of these doubly positive cells (Figure 3B, bottom; Figure S2 in Supplementary Material, right bottom). The numbers of TLR4 and α-SMA doubly positive cells (yellow, merged color by red, and green) in each field (200×) with or without TAK242 treatment were 63.3 ± 6.2 and 18.2 ± 5.7, respectively (Figure 3B, bottom), while the numbers of TLR4 and CYGB doubly positive cells in each field (200×) were 256.0 ± 34.1 and 33.0 ± 7.3, respectively (Figure S2 in Supplementary Material, right bottom). The liver sections of TAK242-treated mice also showed reduced levels of desmin expression after *Sj* infection compared to the non-treated control according to the IHC assay (Figure S3 in Supplementary Material). These results suggest that the activated TLR4 signal pathway in HSCs may contribute to the activation of HSCs and fibrogenesis induced by *Sj* infection. When we stimulated the *in vitro* cultured HSC line with LPS to activate TLR4 signaling, we found that the protein level of α-SMA was upregulated in a LPS-dose-dependent way (Figure 3C; Figure S4A in Supplementary Material). The SEA of *Sj* also triggered TLR4 and α-SMA expression in the *in vitro* cultured HSCs in a dose-dependent way (Figure 3D; Figure S4B in Supplementary Material). Altogether, these results suggest that the TLR4 signal pathway in HSCs contributes to the activation of HSCs during *Sj* infection.

### TLR4 Signaling Regulates the Expression of TGM2

**Figure 4:**
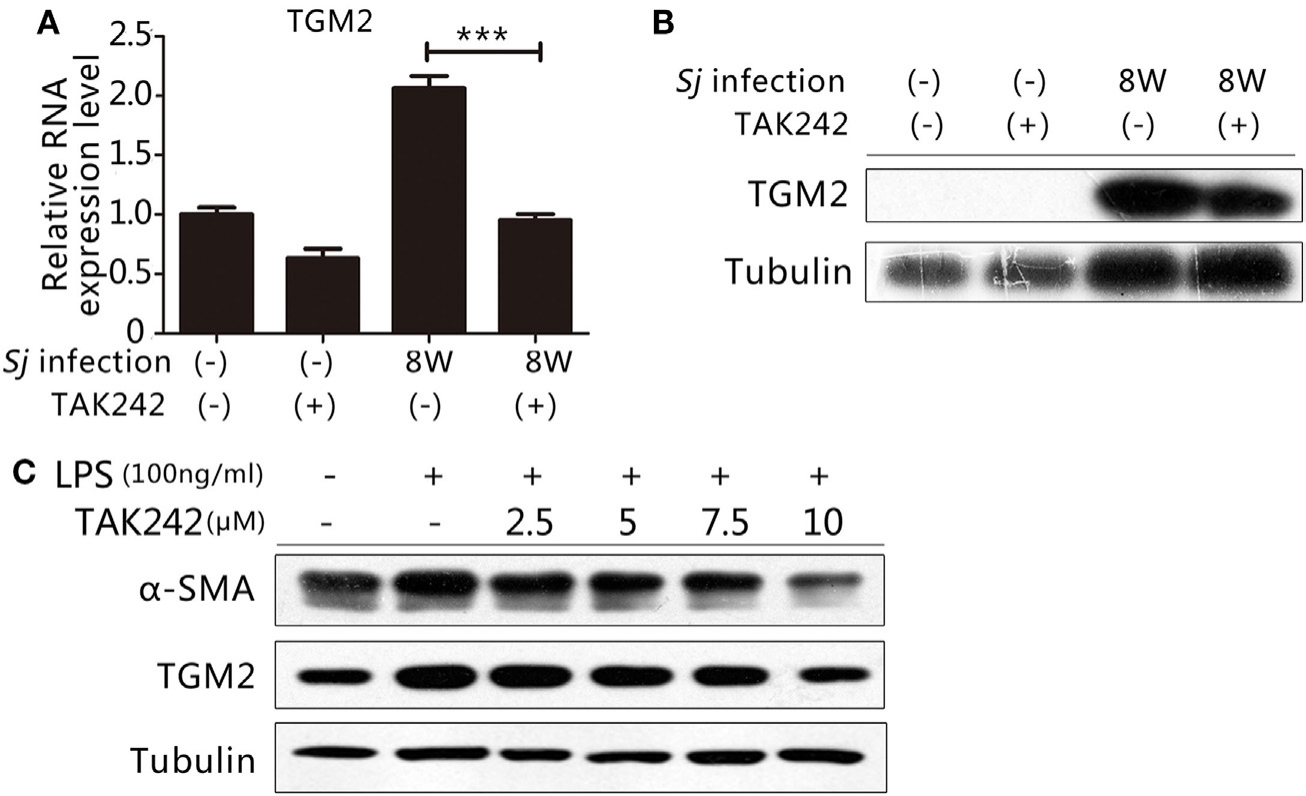
Toll-like receptor (TLR) 4 signaling controlled transglutaminase 2 (TGM2) expression. To determine whether the gene encoding TGM2 is a downstream target of TLR4 signaling, **(A)** the mRNA expression level of Tgm2 in the liver tissue of BALB/c mice post 8-week *Schistosoma japonicum* (*Sj*) infection with or without TAK242 treatment was measured using qPCR; **(B)** the TGM2 protein level of mouse liver homogenates was tested by western blotting. β-Tubulin was used as a loading control; **(C)** *in vitro* cultured hepatic stellate cells exposed to lipopolysaccharide (LPS) with or without TAK242 treatment in different doses, TGM2 and α-SMA protein expression levels were tested by western blotting. β-Tubulin was used as a loading control. All experiments were performed for two or three times. *P ≤ 0.05; **P < 0.01; ***P < 0.001.

Our previous work indicated that TGM2 contributes to liver fibrosis post *Sj* infection (19, 20). Thus, both TGM2 and activation of the TLR4 signal pathway correlated with the development of liver fibrosis during *Sj* infection. As an inflammatory factor, TGM2 might be under the control of TLR4 signaling and involved in the activation of HSCs. The mRNA and protein expression levels of Tgm2 in the 8-week *Sj-*infected mouse liver with TAK242 treatment were significantly reduced compared to those of the non-treated control (Figures 4A,B; Figure S5A in Supplementary Material). In the HSC line, the TGM2 and α-SMA levels both increased after LPS stimulation and were reduced by TAK242 treatment in a dose-dependent way (Figures 4C; Figure S5B in Supplementary Material), thereby indicating that Tgm2 expression is regulated by the TLR4 signal pathway.

### TGM2 Is Involved in Liver Fibrosis during *Sj* Infection As an Upstream Factor That Activates TLR4 Signaling in HSCs

Positive feedback regulations often occur in immune response and immune-related diseases. We examined whether TGM2 might not only act downstream of TLR4 signaling but also act as an upstream activator of the TLR4 signal pathway in our infection model.

**Figure 5:**
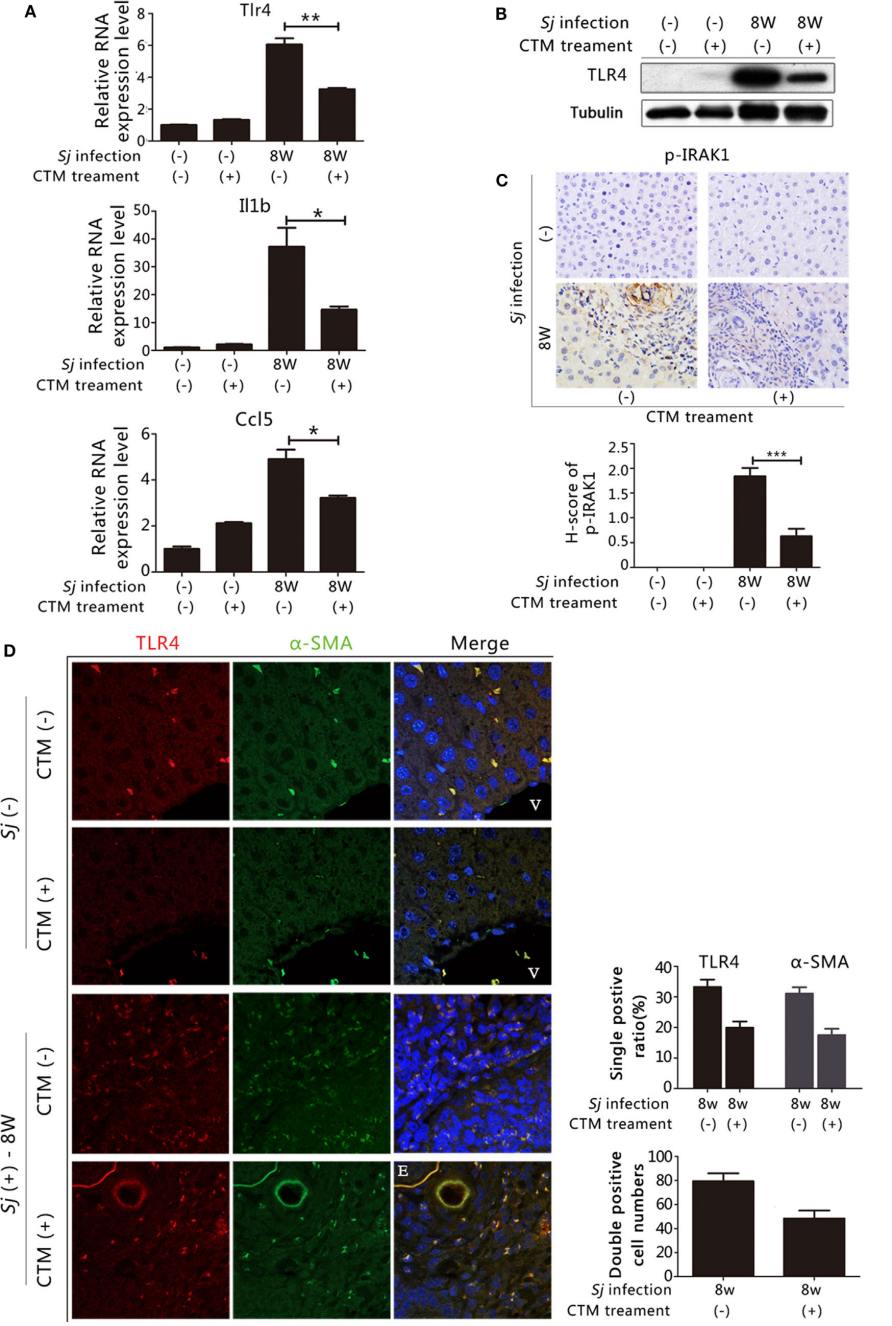

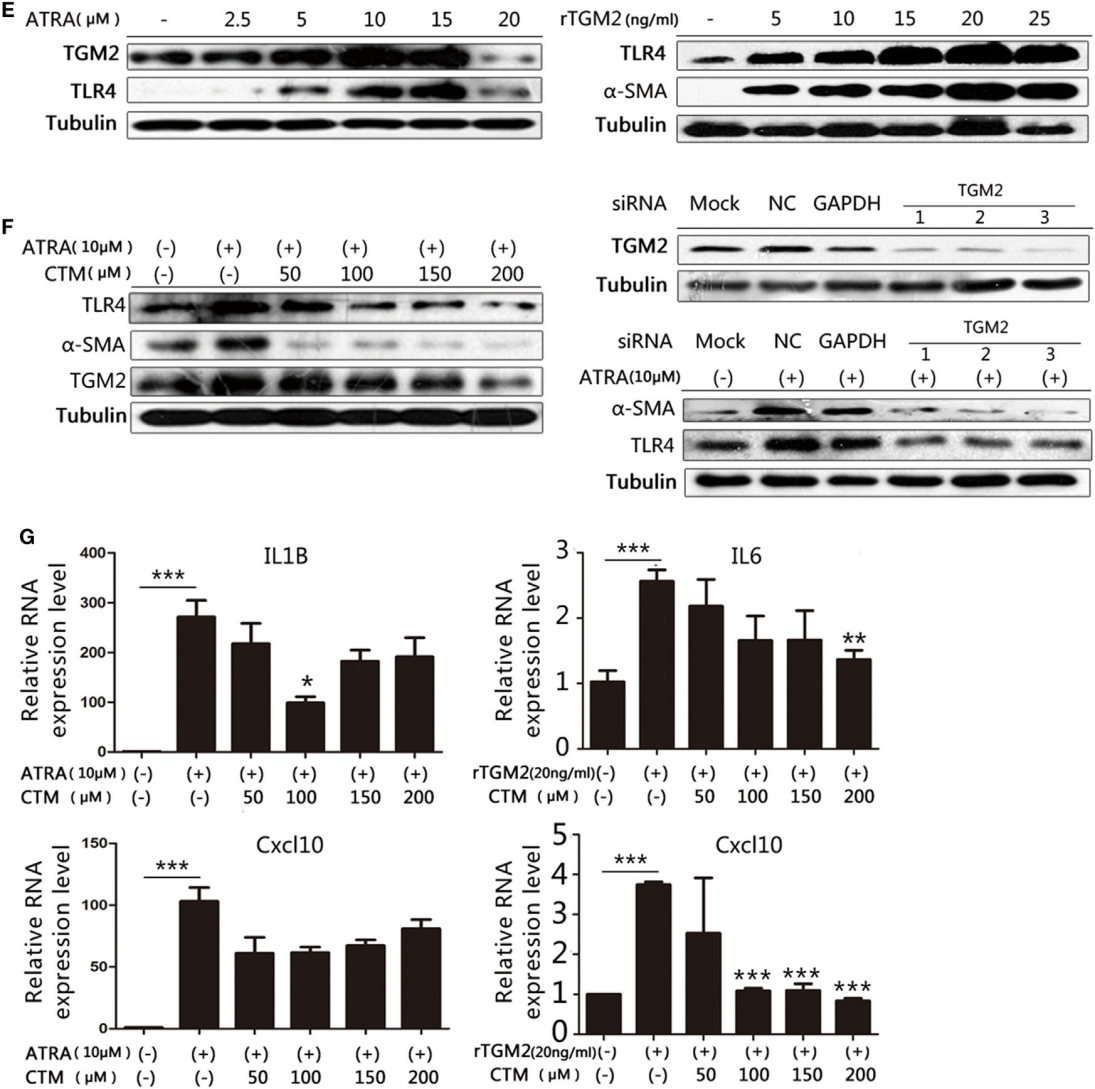
Transglutaminase 2 (TGM2), also acts as an upstream factor that activates TLR4 signaling in hepatic stellate cells (HSCs) and is involved in liver fibrosis during *Schistosoma japonicum* (*Sj*) infection. TGM2 activity in BALB/c mice was inhibited by intraperitoneal injection of cystamine (CTM) from day 3 to day 10 post *Sj* infection. Mice were sacrificed at week 8 post-infection. Non-infected mice with or without CTM treatment served as controls. **(A)** Steady-state RNA expression levels of Tlr4, Il1b, and Ccl5 in the liver were measured through qPCR. **(B)** Protein expression levels of TLR4 were measured by western blotting. **(C)** Mouse livers at indicated time points were fixed in paraformaldehyde, embedded in paraffin, sectioned, and then immunohistochemical (IHC) stained for p-IRAK1 (upper panel). Quantification of the stainings is shown in the bottom panel. **(D)** The expression of TLR4 and α-SMA in the mouse liver were revealed using IF staining (400×). A typical IF result is shown in the left panel, and the ratio or number of cells with indicated positive staining in each field (200×) was calculated and shown in the right panel: top, cells positive for TLR4 or α-SMA; bottom: cells doubly positive for both antigens. **(E)** *In vitro* cultured HSCs were exposed to all-trans-retinoic acid (ATRA) (*left*) or recombinant TGM2 (*right*) at different doses; TLR4 and α-SMA protein expression levels were monitored by western blotting (β-tubulin was used as a loading control). **(F)** With or without CTM at different doses (*left*) or siRNA (*right bottom*) pre-treatment, cultured HSCs triggered by ATRA, TLR4 and α-SMA protein expression levels were revealed by western blotting. The downregulation effect of 3 siRNAs of Tgm2 gene were shown in the right *top* panel. β-tubulin was used as a loading control. **(G)** IL1B, IL6, and Cxcl10 steady-state mRNA expression levels were measured by qPCR. All experiments were performed two or three times. *P ≤ 0.05; **P < 0.01; ***P < 0.001. E, eggs of *Sj*; V, vein in the mice liver.

In our previous work, we found that treating mice with CTM, a TGM2 inhibitor, decreased the levels of TGM2 and liver fibrosis in the 8-week *Sj-*infected mouse liver (19). We observed that the mRNA expression levels of Tlr4, Il1b, and Ccl5 (Figure 5A) as well as the TLR4 protein expression levels were downregulated in CTM-treated mice with respect to the non-treated control (Figure 5B; Figure S11A in Supplementary Material). IHC assay results showed that the level of p-IRAK1, an important cytoplasmic factor of the TLR4-MyD88-dependent pathway, decreased upon CTM treatment (Figure 5C), as p-IRAK1 positive cells were rarely found in the CTM-treated mouse liver sections (Figure 5C). TGM2 can interact with fibronectin (FN) with high affinity (31), and FN extra domain A was identified as an agonist of TLR4 (32). We found that the RNA expression level of Fn1 during *Sj* infection was accordingly increased at weeks 5 and 6, which correlated with the level of expression of Tgm2 and TLR4 pathway effector genes (Figure S6 in Supplementary Material).

We investigated further whether TGM2–TLR4 signaling regulation also operates in HSCs in *Sj*-infected mouse liver. We detected TLR4, α-SMA, and CYGB in the 8-week *Sj* infection mice livers with or without CTM treatment using IF assay. CTM treatment dramatically decreased the levels of TLR4, α-SMA (Figure 5D, *right top*), and CYGB (Figure S7 in Supplementary Material, *top right*). Moreover, the treatment significantly decreased the number of TLR4 and α-SMA doubly positive cells (Figure 5D, *left*) and also TLR4 and CYGB doubly positive cells (Figure S5 in Supplementary Material *left*). The number of TLR4, α-SMA doubly positive cells changed from 79.3 ± 6.7 in every 200× field in the CTM treatment group to 48.4 ± 6.6 in the non-treatment group (Figure 5D, *bottom right*), whereas that of TLR4, CYGB doubly positive cells changed from 256.0 ± 34.1 to 66.4 ± 16.3 (Figure S5 in Supplementary Material, *bottom right*). The IHC assay results showed that the expression level of desmin significantly decreased in the liver sections of the CTM-treated mice after *Sj* infection in comparison with the non-treatment group (Figure S8 in Supplementary Material). Western blotting analysis showed that CTM treatment significantly decreased cleaved caspase 3 (c-casp3) levels in the infected mice livers (Figure S9 in Supplementary Material). Furthermore, CTM-treated mice after *Sj* infection displayed no significant change in egg numbers when compared with the non-treated control group (Figure S10 in Supplementary Material). These results thereby exclude the possibility that CTM treatment triggers caspase 3 cleavage in the host liver and thus triggers apoptosis of HSCs and also rule out that CTM directly acts upon the pathogen during *Sj* infection.

We, further, verified the effect of TGM2 regulation on TLR4 signaling in the cultured HSC line. As Tgm2 gene promoter contained a retinoic acid response element (1.7 kb upstream of the initiation site), all trans-retinoic acid (ARTA) was used to trigger TGM2 expression (33). In cultured HSCs, ATRA at 2.5–15 µM (Figure 5E, *left* and Figure S11B in Supplementary Material) and rTGM2 (Figure 5E, *right* and Figure S11C in Supplementary Material) treatment effectively upregulated TLR4 and α-SMA expression, whereas ATRA treatment at 20 µM downregulated TGM2 and TLR4 (Figure 5E, *left* and Figure S11B in Supplementary Material). The upregulation of TLR4, TGM2, and α-SMA in response to 10 µM ATRA could be reduced by CTM treatment in a dose-dependent manner (Figure 5F, *left* and Figure S11D in Supplementary Material). Moreover, treatment with validated specific siRNAs of Tgm2 (Figure 5F, *bottom right* and Figure S11E in Supplementary Material) confirmed this result (Figure 5F, *top right* and Figure S11F in Supplementary Material). Meanwhile, the RNA expression levels of the IL1B, IL6, and Cxcl10, readouts of TLR4 pathway activation were significantly upregulated with 10 µM ATRA and 20 ng/ml human rTGM2. Almost all of these levels could be lowered by CTM treatment, except for Cxcl10 under ATRA stimulation (Figure 5G).

**Figure 6:**
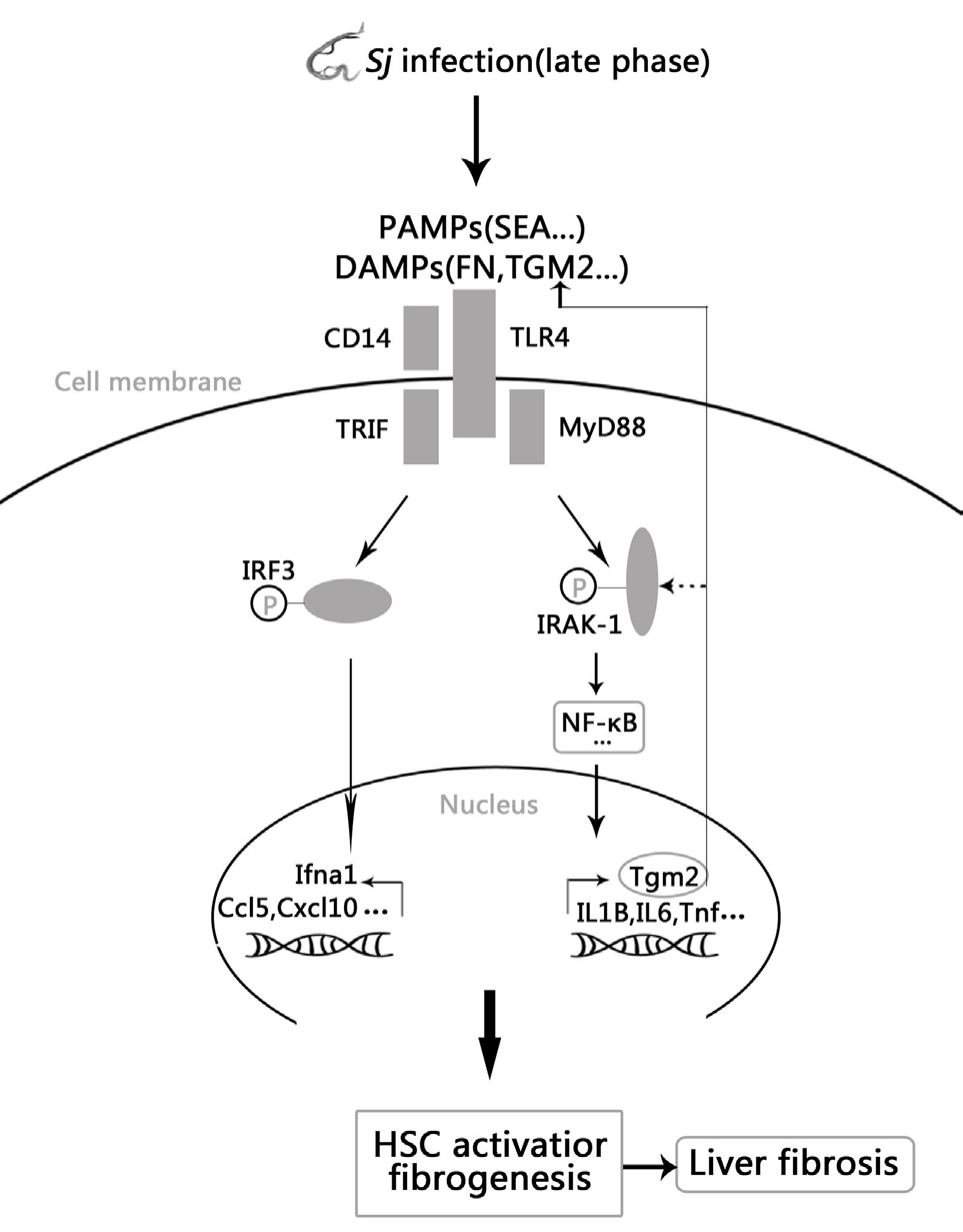
Model of the positive feedback regulation between transglutaminase 2 (TGM2) and TLR4 signaling in hepatic stellate cells (HSCs) activation and liver fibrosis. *Schistosoma japonicum* (*Sj*) infection-activated TLR4 pathway leads to Tgm2 induction, which in turn contributes to HSCs activation and then liver fibrosis. Moreover, upregulated TGM2 positively strengthened TLR4 signaling by enhancing TLR4 activation or possibly by triggering IRAK1 phosphorylation.

Collectively, these results establish a novel positive feedback regulation in which *Sj* infection activated TLR4 signal pathway increases the expression of TGM2, which in turn contributes to HSCs activation through TLR4 pathway and then causes liver fibrosis. Moreover, upregulated TGM2 positively enhances TLR4 signaling by triggering TLR4 ligation or possibly through IRAK1 phosphorylation (Figure 6).

## Discussion

Despite major efforts on this research topic, the mechanisms of liver fibrosis post *Sj* infection remain unclear to date. TLR signaling contributes to the development of chronic liver diseases *via* complex immunopathogenesis (34). TLR4 activation in HSCs is a key step in collagen production and a major mechanism for the development of fibrosis and cirrhosis (11, 14, 35, 36). Activated HSCs can express TLR4 and CD14 and respond to LPS *via* the secretion of pro-inflammatory cytokines (such as IL-6 and IL-8) and several chemokines or adhesion molecules (such as monocyte chemotactic protein 1, macrophage inflammatory protein-2, ICAM-1, VCAM-1, CCL5, and CXCL10) (14, 15, 37, 38). In chimeric C3H/HeJ mice with TLR4 mutation in HSCs, remission of hepatic fibrosis induced by exposure to LPS indicated a main role for HSCs in hepatic inflammation and fibrosis (11, 39). In this work, we show that the transcription of Tlr4 and Cd14 is upregulated in the liver of *Sj*-infected mice, and that suppression of TLR4 signaling by TAK242 decreases the extent of hepatic fibrosis induced by *Sj* infection. We also observed the consistent correlation of the expressions of TLR4, α-SMA, CYGB, and desmin in the HSCs of *Sj*-infected liver with or without TAK242 treatment through the IF or IHC method. Importantly, under SEA of *Sj* or LPS stimulation, TLR4 and α-SMA levels in cultured HSCs were upregulated in a dose-dependent manner. As α-SMA is a marker of activated HSCs in mice (30), these results indicate that *Sj*-activated TLR4 signaling induces HSCs activation and then liver fibrogenesis. Compared with wild-type mice, Tlr4 (−/−) mice showed a higher survival ratio and a higher extent of liver fibrosis, but the difference was not significant (data not shown). Zhang et al. (40) have reported that Tlr4(−/−) mice display reduced levels of IL-12, IFN-γ, and IL-4 in dermal tissues and retroauricular draining lymph nodes 4 days after *Sj* infection as well as increased egg burden and decreased T cell activation at week 6. Durães et al. (41) concluded that TLR4 is a key receptor involved in *Sm* tegument induction of pro-inflammatory cytokines. These results indicate that TLR4 signaling is needed for the host’s protective response at an early stage of *Schistosoma* infection. This early protective function of TLR4 renders the use of Tlr4 (−/−) mice unsuitable for the study of liver fibrosis at the late stage of *Sj* infection. As a first step, we therefore used the TLR4 pathway inhibitor TAK242 at week 4, when *Sj* enters the liver for 4 weeks. Of note, as we cannot formally exclude a possible direct action of the TAK242 inhibitor on *Sj*, the results obtained with this inhibitor will need to be confirmed in future work either using a conditional knockout of TLR4 in HSCs, or using the chimeric C3H/HeJ HSCs mouse model (11, 39).

Transglutaminase 2, a multifunctional enzyme, is reportedly involved in liver fibrosis (18, 19). We also provided evidence that TGM2 regulates TGF-β1 of *Sj* origin and IL-13 of the host and documented the important involvement of TGM2 in liver fibrosis during *Sj* infection in previous studies (19, 20). TGM2 and TLR4 levels display a similar pattern of expression changes that correlates with the extent of liver fibrosis post *Sj* infection. Seki et al. (11) reported that TLR4-enhanced TGF-β1 signaling and HSCs activation promote hepatic fibrosis, which prompted us to explore the relationship between TGM2 and TLR4 signaling in liver fibrosis post *Sj* infection. Brown (42) reviewed that NF-κB pathway activation upregulates TGM2 level in the breast cancer, and Kiziltas (34) reported that NF-κB pathway activation is the common downstream pathway of TLR4 signal pathway activation in the liver. Based on these data, we hypothesized that TLR4 signaling might act upstream of TGM2 during *Sj* infection. As expected, the RNA and protein expression levels of Tgm2 significantly decrease in the liver of 8-week *Sj*-infected mice treated with the TLR4 inhibitor TAK242, and the expression levels of TGM2 and α-SMA increase *in vitro* in cultured HSCs under LPS stimulation. Moreover, the increased levels are dose-dependently lowered by TAK242 treatment. These results suggest that Tgm2 is a downstream target of TLR4 signaling during *Sj* infection. The mechanisms by which TLR4 regulates Tgm2 expression will need to be clarified in future experiments.

Positive feedback regulation usually occurs in immune-related diseases. In the present research, we found that TGM2 expression is not only regulated through the TLR4 pathway but also its protein product acts upstream to activate this pathway. The TGM2 inhibitor CTM decreased the level of expression of TLR4, of the TLR4 pathway-dependent effector cytokines, and levels of p-IRAK1, a cytoplasmic adaptor of the TLR4-MyD88-dependent pathway in *Sj*-infected mice liver. However, we found that p-IRF3 levels were also upregulated in HSCs, suggesting an involvement of the TLR4/MyD88-independent pathway (data not shown). Petrasek et al. (43) reported that high levels of p-IRF3 in parenchymal cells of the liver exert protective effect against liver injury in alcoholic mice. In-depth studies will be performed to assess the effect of CTM treatment or Tgm2 downregulation on p-IRAK-1 and p-IRF3 expression. IF results establish that the TGM2-TLR4 pathway is regulated in the HSCs of *Sj*-infected mouse liver. Furthermore, we have confirmed that this regulation also takes place in *in vitro* cultured HSCs. Treatment with ATRA (a trigger of TGM2 expression), and rTGM2 effectively upregulated TGM2, TLR4, and α-SMA, and the upregulation was alleviated by CTM or specific siRNA of TGM2. The transcription levels of IL1B, IL6, and Cxcl10 were also upregulated by the ATRA treatment. Taken together, our results indicate that a novel self-reinforcing molecular feedback loop takes place between TGM2 and the TLR4 pathway in HSCs. Given that TLR4 expression in mice liver without treatment is very low or absent (34), this feedback loop should not exist in normal mice.

Using *Myd88*-deficient mice, Seki et al. (11) concluded that the TLR4-MyD88–NF-κB axis is linked to hepatic fibrosis induced by elevated LPS levels. However, TLR4-MyD88-independent activation by damage-associated molecular patterns (DAMPs) is central to the inflammatory process in ischemia–reperfusion lesions (44, 45). In our infection model, the following indirect evidence suggests that TLR4 signaling is possibly MyD88-dependent: there is a significant increase of the expression levels of the TLR4/MyD88-dependent effector cytokine genes, Tnf, and Il1ß at week 6 when liver fibrosis is acute and then at week 8 when liver fibrosis is advanced after *Sj* infection. However, the levels of CXCL10 and CCL5, readout cytokines of the MyD88-independent TLR4 pathway increased at week 5 when *Sj* eggs induce granuloma and then at week 6 when *Sj* eggs induce acute liver damage. Only the RNA levels of IL1B or IL6 but not those of Cxcl10 were decreased in HSCs after CTM and ATRA treatment.

Cystamine treatment of mice during the early stage of *Sj* infection (from day 3 to 10) alleviates the extent of liver fibrosis but the treatment during the late stage (from week 5 to 6 or from week 6 to 8) did not have this effect (19). The early inhibition role might persist in some cells because TGM2 might affect CD8+ memory T-cell generation as reported by Kim et al. (46). However, the exact mechanism needs to be elucidated. Tgm2(−/−) mice were not used in this study to verify the results because of the inconsistent effect of CTM treatment in the early and late stages of liver fibrosis.

In general, the positive feedback regulation between TGM2 and TLR4 signaling in HSCs correlated with liver fibrosis post *Sj* infection. It is, however, unclear at this stage as to which stimulus actually triggers the TLR4 pathway during *Sj* infection. Results showed that the level of FN was correlated with the level of TGM2, TLR4, and the extent of liver fibrosis during *Sj* infection. SEA of *Sj* and rTGM2 induce TLR4 and α-SMA expression in cultured HSCs in a dose-dependent manner. Thus, potential TLR4 ligands during *Sj* infection include pathogen associated molecular patterns such as the components of SEA from the parasite, possibly some danger associated molecular patterns (DAMPs), e.g., FN and/or TGM2 from the host. The novel self-reinforcing molecular feedback loop between the TLR4 pathway and TGM2 may represent a potential new target in *Sj* infection-induced liver fibrosis and even other related liver diseases. Future studies will reveal whether the TGM2/TLR4 pathway axis is relevant in other inflammatory diseases.

## Ethics Statement

Six- to eight-week-old female BABL/c mice (Experimental Animal Center of Sun Yat-Sen University, Guangzhou, China) were maintained according to the guidelines and were approved to be appropriate and humane by Institutional Animal Care and Use Committee of Guangzhou Medical University.

## Author Contributions

ZW, XJ, JT, and GL contributed equally to this work. ZW, and XJ performed the research; ZL, ZW, XJ, JT, and DF designed the research; JT, ZW, XJ, GL, LX, MW, and CL contributed essential reagents or tools; ZW analyzed the data; ZW, ZL, and JT wrote the paper; ZL and JT revised the paper.

## Conflict of Interest Statement

The authors declare that the research was conducted in the absence of any commercial or financial relationships that could be construed as a potential conflict of interest.

## References

[1] Colley DG Bustinduy AL Secor WE King CH. Human schistosomiasis. Lancet (2014) 383(9936):2253–64. 10.1016/S0140-6736(13)61949-2 24698483 PMC4672382

[2] Friedman SL. Evolving challenges in hepatic fibrosis. Nat Rev Gastroenterol Hepatol (2010) 7(8):425–36. 10.1038/nrgastro.2010.97 20585339

[3] Friedman SL. Mechanisms of hepatic fibrogenesis. Gastroenterology (2008) 134(6):1655–69. 10.1053/j.gastro.2008.03.003 18471545 PMC2888539

[4] Kisseleva T Brenner DA. Role of hepatic stellate cells in fibrogenesis and the reversal of fibrosis. J Gastroenterol Hepatol (2007) 22(Suppl 1):S73–8. 10.1111/j.1440-1746.2006.04658.x 17567473

[5] Seki E Schnabl B. Role of innate immunity and the microbiota in liver fibrosis: crosstalk between the liver and gut. J Physiol (2012) 590(3):447–58. 10.1113/jphysiol.2011.219691 22124143 PMC3379693

[6] Nakamoto N Kanai T. Role of toll-like receptors in immune activation and tolerance in the liver. Front Immunol (2014) 5:221. 10.3389/fimmu.2014.00221 24904576 PMC4032908

[7] Petrasek J Csak T Szabo G. Toll-like receptors in liver disease. Adv Clin Chem (2013) 59:155–201. 10.1016/B978-0-12-405211-6.00006-1 23461136

[8] Hritz I Mandrekar P Velayudham A Catalano D Dolganiuc A Kodys K The critical role of toll-like receptor (TLR) 4 in alcoholic liver disease is independent of the common TLR adapter MyD88. Hepatology (2008) 48(4):1224–31. 10.1002/hep.22470 18792393 PMC7137387

[9] Rivera CA Adegboyega P van Rooijen N Tagalicud A Allman M Wallace M. Toll-like receptor-4 signaling and Kupffer cells play pivotal roles in the pathogenesis of non-alcoholic steatohepatitis. J Hepatol (2007) 47:571–9. 10.1016/j.jhep.2007.04.019 17644211 PMC2094119

[10] Csak T Velayudham A Hritz I Petrasek J Levin I Lippai D Deficiency in myeloid differentiation factor-2 and toll-like receptor 4 expression attenuates nonalcoholic steatohepatitis and fibrosis in mice. Am J Physiol Gastrointest Liver Physiol (2011) 300(3):G433–41. 10.1152/ajpgi.00163.2009 21233280 PMC3302188

[11] Seki E De Minicis S Osterreicher CH Kluwe J Osawa Y Brenner DA TLR4 enhances TGF-beta signaling and hepatic fibrosis. Nat Med (2007) 13(11):1324–32. 10.1038/nm1663 17952090

[12] Wang B Trippler M Pei R Lu M Broering R Gerken G Toll-like receptor activated human and murine hepatic stellate cells are potent regulators of hepatitis C virus replication. J Hepatol (2009) 51(6):1037–45. 10.1016/j.jhep.2009.06.020 19716616

[13] Aoyama T Paik YH Seki E Toll-like receptor signaling and liver fibrosis. Gastroenterol Res Pract (2010) 2010:192543 10.1155/2010/192543 20706677 PMC2913673

[14] Paik YH Schwabe RF Bataller R Russo MP Jobin C Brenner DA Toll-like receptor 4 mediates inflammatory signaling by bacterial lipopolysaccharide in human hepatic stellate cells. Hepatology (2003) 37(5):1043–55. 10.1053/jhep.2003.50182 12717385

[15] Yang L Seki E. Toll-like receptors in liver fibrosis: cellular crosstalk and mechanisms. Front Physiol (2012) 3:138. 10.3389/fphys.2012.00138 22661952 PMC3357552

[16] van Stijn CM Meyer S van den Broek M Bruijns SC van Kooyk Y Geyer R *Schistosoma mansoni* worm glycolipids induce an inflammatory phenotype in human dendritic cells by cooperation of TLR4 and DC-SIGN. Mol Immunol (2010) 47(7–8):1544–52. 10.1016/j.molimm.2010.01.014 20170964

[17] Nurminskaya MV Belkin AM. Cellular functions of tissue transglutaminase. Int Rev Cell Mol Biol (2012) 294:1–97. 10.1016/B978-0-12-394305-7.00001-X 22364871 PMC3746560

[18] Tatsukawa H Furutani Y Hitomi K Kojima S. Transglutaminase 2 has opposing roles in the regulation of cellular functions as well as cell growth and death. Cell Death Dis (2016) 7(6):e2244. 10.1038/cddis.2016.150 27253408 PMC5143380

[19] Tang J Zhu X Zhao J Fung M Li Y Gao Z Tissue transglutaminase-regulated transformed growth factor-β1 in the parasite links *Schistosoma japonicum* infection with liver fibrosis. Mediators Inflamm (2015) 2015:659378. 10.1155/2015/659378 26199461 PMC4493306

[20] Tang J Huang H Ji X Zhu X Li Y She M Involvement of IL-13 and tissue transglutaminase in liver granuloma and fibrosis after *Schistosoma japonicum* infection. Mediators Inflamm (2014) 2014:753483. 10.1155/2014/753483 25110399 PMC4106180

[21] Kawabe K Takano K Moriyama M Nakamura Y. Lipopolysaccharide-stimulated transglutaminase 2 expression enhances endocytosis activity in the mouse microglial cell line BV-2. Neuroimmunomodulation (2015) 22(4):243–9. 10.1159/000365484 25301694

[22] Sarang Z Köröskényi K Pallai A Duró E Melino G Griffin M Transglutaminase 2 null macrophages respond to lipopolysaccharide stimulation by elevated proinflammatory cytokine production due to an enhanced αvβ3 integrin-induced Src tyrosine kinase signaling. Immunol Lett (2011) 138(1):71–8. 10.1016/j.imlet.2011.03.004 21420434

[23] Takano K Shiraiwa K Moriyama M Nakamura Y. Transglutaminase 2 expression induced by lipopolysaccharide stimulation together with NO synthase induction in cultured astrocytes. Neurochem Int (2010) 57(7):812–8. 10.1016/j.neuint.2010.08.019 20817067

[24] Falasca L Farrace MG Rinaldi A Tuosto L Melino G Piacentini M. Transglutaminase type II is involved in the pathogenesis of endotoxic shock. J Immunol (2008) 180:2616–24. 10.4049/jimmunol.180.4.2616 18250473

[25] Matic I Sacchi A Rinaldi A Melino G Khosla C Falasca L Characterization of transglutaminase type II role in dendritic cell differentiation and function. J Leukoc Biol (2010) 88(1):181–8. 10.1189/jlb.1009691 20371597 PMC3210574

[26] Luciani A Villella VR Esposito S Gavina M Russo I Silano M Targeting autophagy as a novel strategy for facilitating the therapeutic action of potentiators on ΔF508 cystic fibrosis transmembrane conductance regulator. Autophagy (2012) 8(11):1657–72. 10.4161/auto.21483 22874563 PMC3494594

[27] Budwit-Novotny DA McCarty KS Cox EB Soper JT Mutch DG Creasman WT Immunohistochemical analyses of estrogen receptor in endometrial adenocarcinoma using amonoclonal antibody. Cancer Res (1986) 46(10):5419–25. 3756890

[28] Matsunaga N Tsuchimori N Matsumoto T Ii M. TAK-242 (resatorvid), a small-molecule inhibitor of Toll-like receptor (TLR) 4 signaling, binds selectively to TLR4 and interferes with interactions between TLR4 and its adaptor molecules. Mol Pharmacol (2011) 79(1):34–41. 10.1124/mol.110.068064 20881006

[29] Motoyama H Komiya T Thuy le TT Tamori A Enomoto M Morikawa H Cytoglobin is expressed in hepatic stellate cells, but not in myofibroblasts, in normal and fibrotic human liver. Lab Invest (2014) 94(2):192–207. 10.1038/labinvest.2013.135 24296877

[30] Troeger JS Mederacke I Gwak GY Dapito DH Mu X Hsu CC Deactivation of hepatic stellate cells during liver fibrosis resolution in mice. Gastroenterology (2012) 143(4):1073–83.e22. 10.1053/j.gastro.2012.06.036 22750464 PMC3848328

[31] Cardoso I Østerlund EC Stamnaes J Iversen R Andersen JT Jørgensen TJ Dissecting the interaction between transglutaminase 2 and fibronectin. Amino Acids (2017) 49(3):489–500. 10.1007/s00726-016-2296-y 27394141

[32] Julier Z Martino MM de Titta A Jeanbart L Hubbell JA. The TLR4 agonist fibronectin extra domain A is cryptic, exposed by elastase-2; use in a fibrin matrix cancer vaccine. Sci Rep (2015) 5:8569. 10.1038/srep08569 25708982 PMC4338432

[33] Nagy L Thomázy VA Saydak MM Stein JP Davies PJ. The promoter of the mouse tissue transglutaminase gene directs tissue-specific, retinoid-regulated and apoptosis-linked expression. Cell Death Differ (1997) 4(7):534–47. 10.1038/sj.cdd.4400290 14555966

[34] Kiziltas S. Toll-like receptors in pathophysiology of liver diseases. World J Hepatol (2016) 8(32):1354–69. 10.4254/wjh.v8.i32.1354 27917262 PMC5114472

[35] Mencin A Kluwe J Schwabe RF. Toll-like receptors as targets in chronic liver diseases. Gut (2009) 58(5):704–20. 10.1136/gut.2008.156307 19359436 PMC2791673

[36] Bataller R Brenner DA. Liver fibrosis. J Clin Invest (2005) 115(2):209–18. 10.1172/JCI24282 15690074 PMC546435

[37] Kesar V Odin JA Toll-like receptors and liver disease. Liver Int (2014) 34(2):184–96. 10.1111/liv.12315 24118797

[38] Chen Y Sun R. Toll-like receptors in acute liver injury and regeneration. Int Immunopharmacol (2011) 11(10):1433–41. 10.1016/j.intimp.2011.04.023 21601014

[39] Guo J Friedman SL. Toll-like receptor 4 signaling in liver injury and hepatic fibrogenesis. Fibrogenesis Tissue Repair (2010) 21(3):21. 10.1186/1755-1536-3-21 20964825 PMC2984459

[40] Zhang M Gao Y Du X Zhang D Ji M Wu G. Toll-like receptor (TLR) 2 and TLR4 deficiencies exert differential in vivo effects against *Schistosoma japonicum*. Parasite Immunol (2011) 33(4):199–209. 10.1111/j.1365-3024.2010.01265.x 21392041

[41] Durães FV Carvalho NB Melo TT Oliveira SC Fonseca CT. IL-12 and TNF-alpha production by dendritic cells stimulated with *Schistosoma mansoni* schistosomula tegument is TLR4- and MyD88-dependent. Immunol Lett (2009) 125(1):72–7. 10.1016/j.imlet.2009.06.004 19539649

[42] Brown KD. Transglutaminase 2 and NF-κB: an odd couple that shapes breast cancer phenotype. Breast Cancer Res Treat (2013) 137(2):329–36. 10.1007/s10549-012-2351-7 23224146

[43] Petrasek J Dolganiuc A Csak T Nath B Hritz I Kodys K Interferon regulatory factor 3 and type I interferons are protective in alcoholic liver injury in mice by way of crosstalk of parenchymal and myeloid cells. Hepatology (2011) 53(2):649–60. 10.1002/hep.24059 21274885 PMC3069538

[44] Schwabe RF Seki E Brenner DA Toll-like receptor signaling in the liver. Gastroenterology (2006) 130(6):1886–900. 10.1053/j.gastro.2006.01.038 16697751

[45] Chang WJ Toledo-Pereyra LH. Toll-like receptor signaling in liver ischemia and reperfusion. J Invest Surg (2012) 25(4):271–7. 10.3109/08941939.2012.687802 22853814

[46] Kim JH Hong JM Jeong EM Lee WJ Kim HR Kang JS Lack of transglutaminase 2 diminished T-cell responses in mice. Immunology (2014) 142(3):506–16. 10.1111/imm.12282 24628083 PMC4080966

