# Supplementary material for: Positive Feedback Regulation between Transglutaminase 2 and Toll-Like Receptor 4 Signaling in Hepatic Stellate Cells Correlates with Liver Fibrosis Post Schistosoma japonicum Infection
Source: Front Immunol. 2017 Dec 13;8:1808. doi: 10.3389/fimmu.2017.01808 (PMC5733538; doi:10.3389/fimmu.2017.01808)
Supplement: Supplementary file 1 [file data_sheet_1.PDF]

Supplementary Materials

Positive feedback regulation between transglutaminase 2 and TLR4 signaling in hepatic stellate cells correlates with liver fibrosis post *Schistosoma japonicum* infection

Wen Zhencheng[1], Ji Xiaofang[1], Tang Juanjuan[1], Lin Guiying[1], Xiao Linzhuo[1], Liang Cuiying[1], Wang Manni[1], Su Fang[1], Ferrandon Dominique[1,2]*, Li Zi[1]*

[1] Sino-French Hoffmann Institute, Guangzhou Medical University, Guangzhou, P. R. China.
[2] Université de Strasbourg, RIDI UPR9022 du CNRS, F-67000 Strasbourg France

*** Correspondence:**
Dr. Dominique Ferrandon
D.Ferrandon@ibmc-cnrs.unistra.fr
Dr. Zi Li
lizi1002@gzhmu.edu.cn

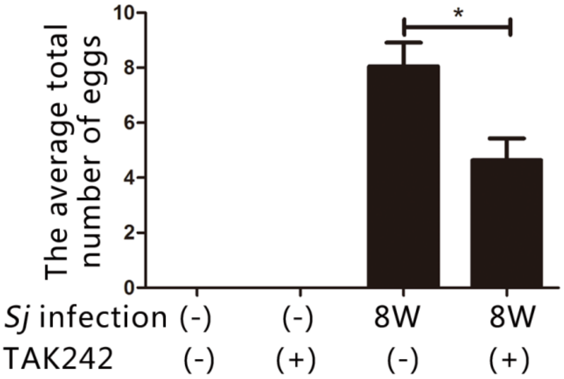

Figure S1 Suppression of TLR4 signaling with TAK242 treatment lowered the *Sj* egg load post *Sj* infection. Liver tissues were fixed and stained with sirius red. The average total number of eggs in five fields (200×) of every section was counted and shown.

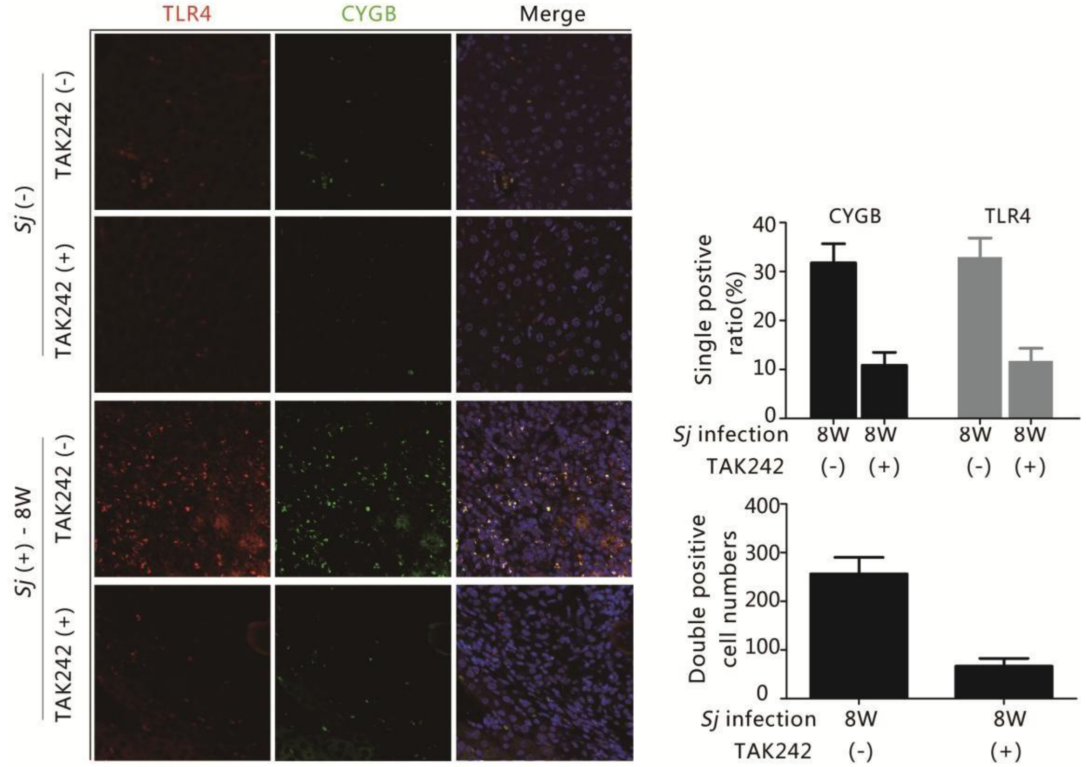

Figure S2. Correlation of TLR4 and CYGB appeared in *Sj*-infected liver cells with or without TAK242 treatment. (Left) The percentage of cells with TLR4 or CYGB positive immunofluorescence (IF) staining in the mouse liver section (400×) of BALB/c mice post *Sj* infection with or without TAK242 treatment was determined. The typical IF result is shown. (Right) The positive expression ratio of TLR4 or CYGB single positive cells in the total cells of mouse liver sections at 400× magnification was measured (*top*), and the total numbers of cells with TLR4 and CYGB double positive were calculated and shown (*bottom*).

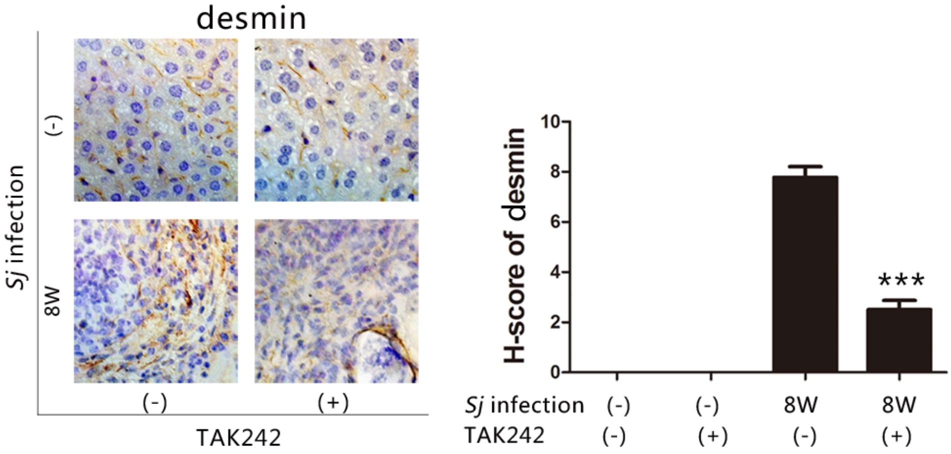

Figure S3. **TAK242 treatment decreased the cells' number with desmin positive staining by IHC.** The typical IHC staining section (400×) of desmin in the liver of BALB/c mice with or without TAK242 treatment were shown in the left panel, and the modified H-score results were shown in the right panel. Data were presented as mean ±SD.

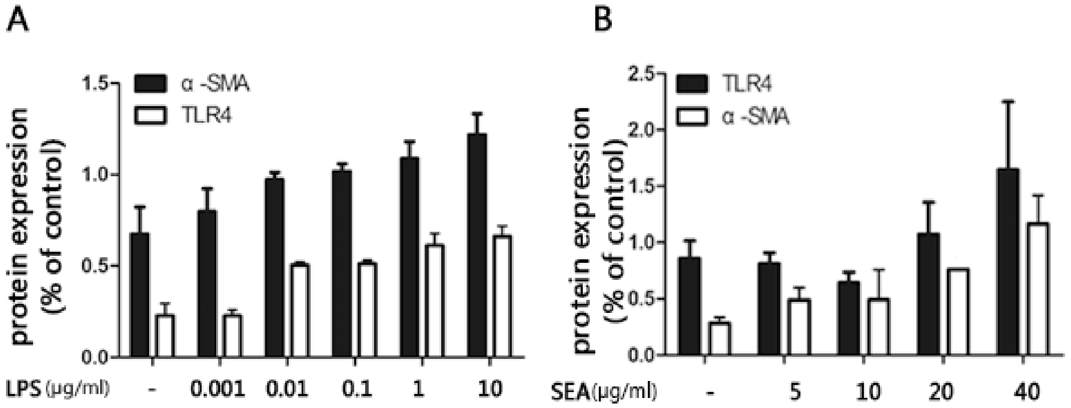

Figure S4. Correlation between TLR4 and α-SMA expression after exposure of cultured HSCs to LPS or *Sj* SEA. In vitro cultured HSCs were exposed to LPS(A) or SEA(B) at indicated doses; TLR4 and α-SMA protein expression levels were monitored by Western blotting. β-Tubulin was used as a loading control. The quantification of the immunoreactive bands of TLR4 and α-SMA (% of control) was shown.

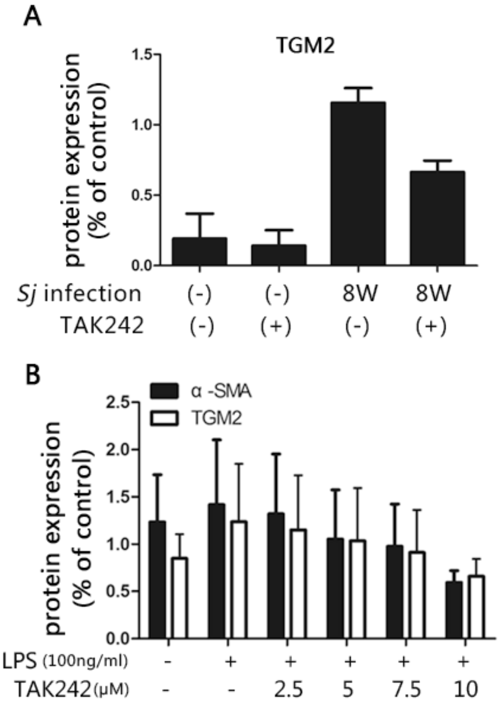

Figure S5. Suppression of TLR4 signaling by TAK242 treatment decreases TGM2 and α-SMA expression. (A) The TGM2 protein level in the liver tissue of BALB/c mice post 8-week *Sj* infection with or without TAK242 treatment was tested by Western blotting;(B) in vitro cultured HSCs exposed to LPS with or without TAK242 treatment in different doses, TGM2 and α-SMA protein expression levels were tested by Western blotting.β-Tubulin was used as a loading control. The quantification of the immunoreactive bands of TGM2 and α-SMA (% of control) was shown.

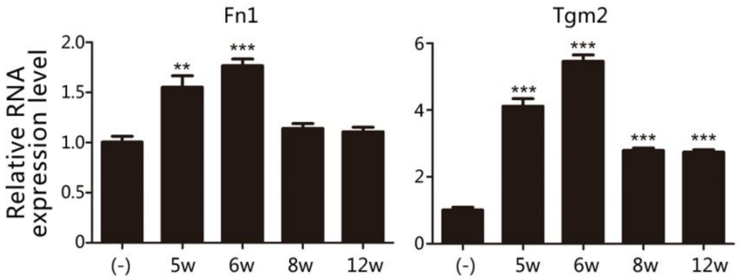

Figure S6. The RNA expression level of Fn1 was consistent with the expression level of Tgm2 post *Sj* infection.The mRNA expression levels of Fibronectin 1 (Fn1) and Tgm2 in the liver tissue of BALB/c mice were shown by qPCR. Gapdh was detected as an internal control.

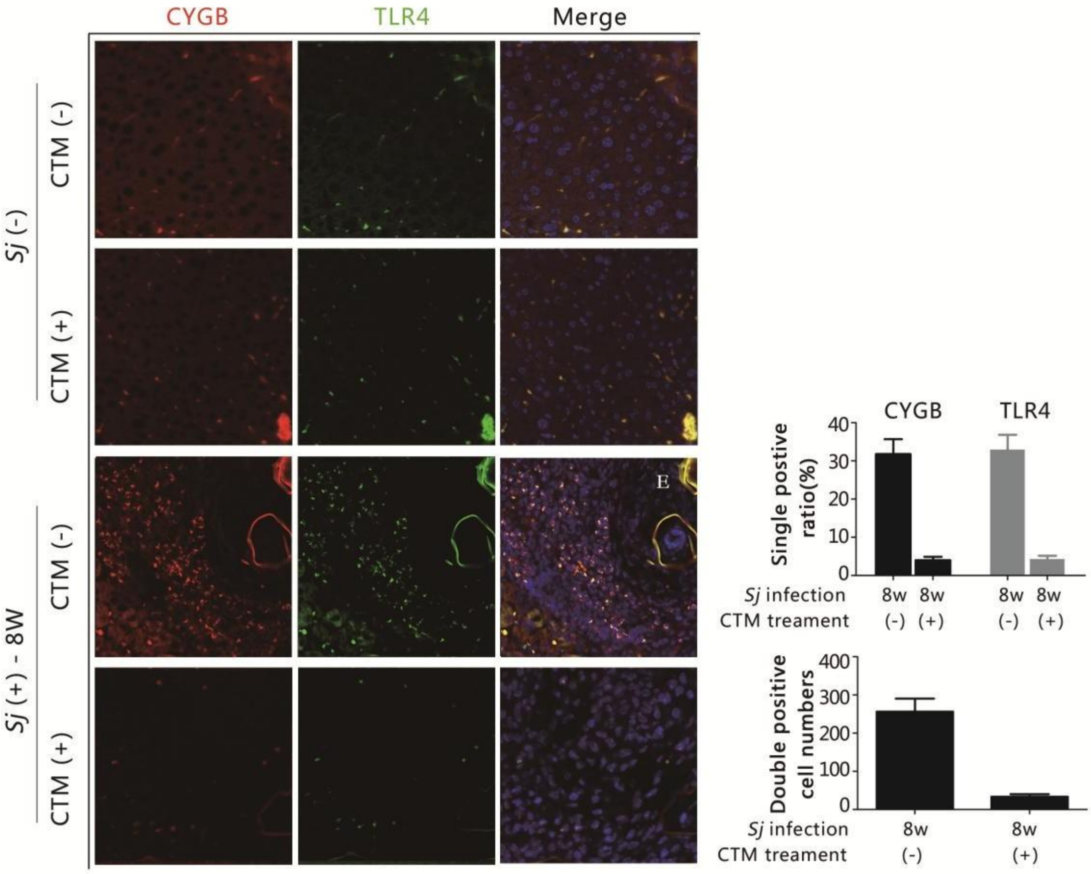

Figure S7. Correlation of TLR4 and CYGB appeared in *Sj*-infected liver cells with or without CTM treatment. The ratio of cells with TLR4 or CYGB single positive, and the total number of cells with TLR4 and CYGB double positive using IF staining (400×) in the mice liver post *Sj* infection with or without CTM treatment were determined. The typical IF result was shown in the left panel, and the ratio or number of cells with indicated positive staining were calculated and shown in the right panel.

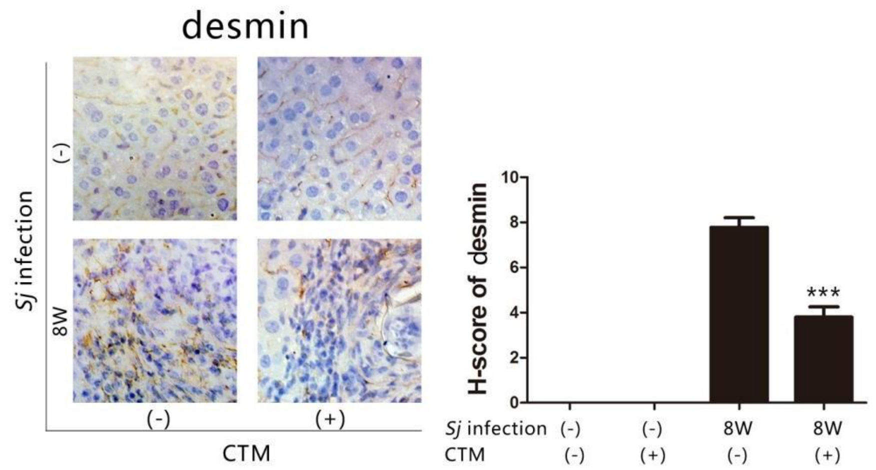

Figure S8. CTM treatment decreased the cell number with desmin positive staining by IHC. The typical IHC staining(400×) section of desmin in the liver of BALB/c mice with or without CTM treatment were shown in the left panel, and the modified H-score results were shown in the right panel. Data were presented as mean ±SD.

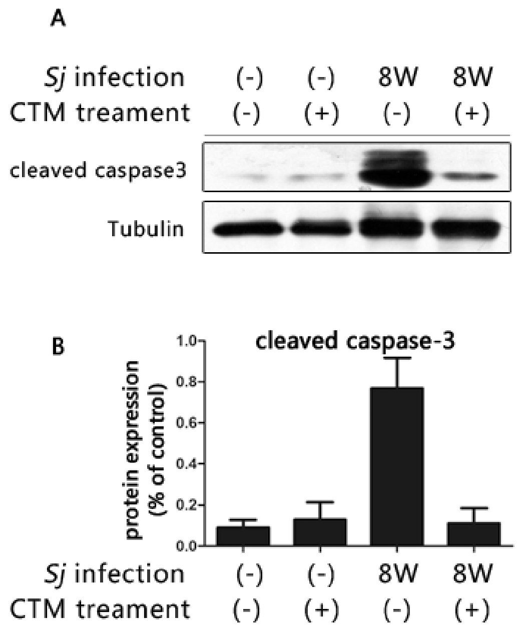

Figure S9. CTM treatment lowered the protein expression level of cleaved caspase3 in the mice liver post *Sj* infection. A. Cleaved caspase3 protein levels of mice liver homogenates with or without CTM treatment were tested by Western blotting. β-Tubulin was used as loading control. B. The quantification of the immunoreactive bands of cleaved caspase3 protein (% of control) was shown.

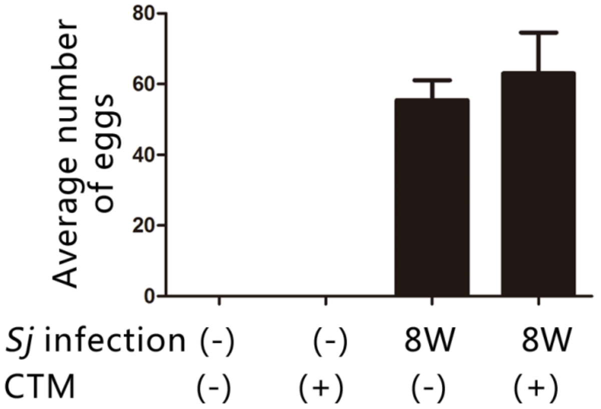

Figure S10. **Suppression of TGM2 activity with CTM didn't change *Sj* egg load post *Sj* infection.** Liver tissues were fixed and stained with sirius red. The average total number of eggs in five fields (200 ×) of every section was counted and shown.

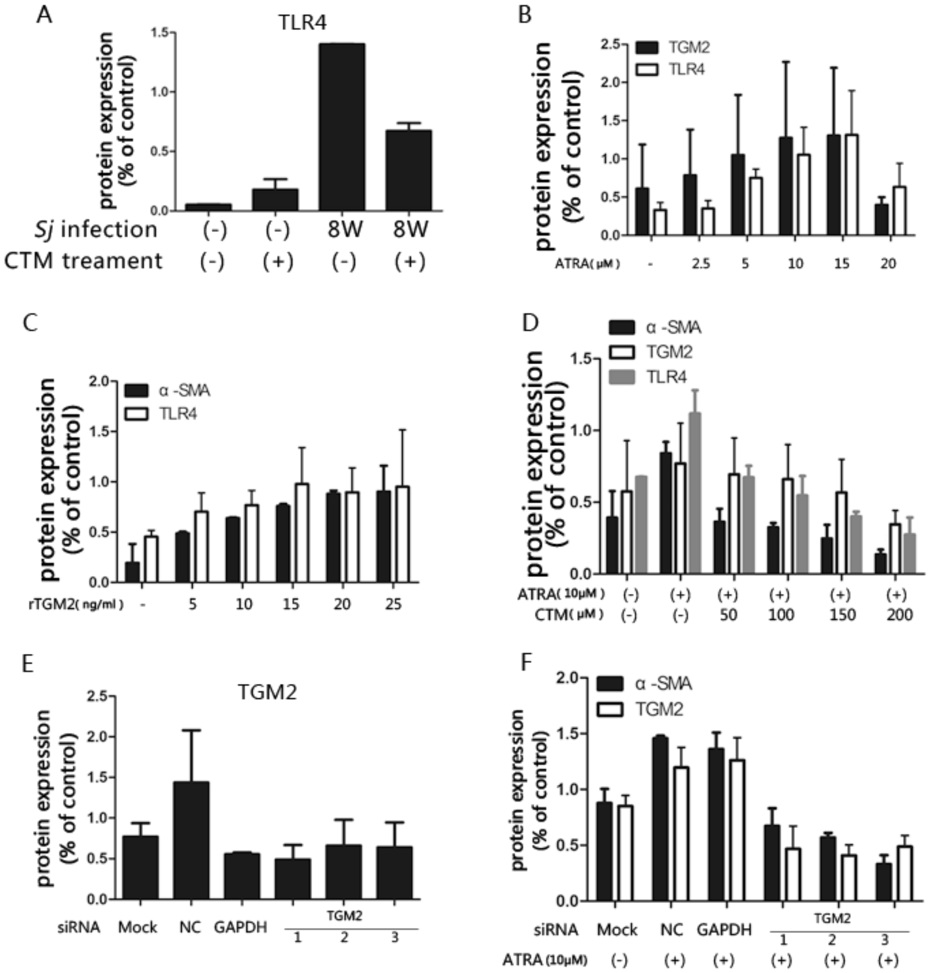

Figure S11. TGM2 as an upstream factor activates TLR4 signaling in HSCs and is involved in liver fibrosis during *Sj* infection. (A) The TLR4 protein level of mouse liver homogenates with or without CTM treatment was tested by Western blotting. *In vitro* cultured HSCs were exposed to ATRA (B) or recombinant TGM2 (C) at indicated doses, TLR4 and α-SMA protein expression levels were monitored by Western blotting; (D) With or without CTM at different doses pre-treatment, cultured HSCs triggered by ATRA, TLR4 and α-SMA protein

expression levels were revealed by Western blotting; (E,F)siRNA pre-treatment, cultured HSCs triggered by ATRA, TLR4 and α-SMA protein expression levels were revealed by Western blotting. β-Tubulin was used as a loading control. The quantification of the immunoreactive bands of target proteins (% of control) was shown.
